# Role of Toll-Like Receptor 4 in Colorectal Carcinogenesis: A Meta-Analysis

**DOI:** 10.1371/journal.pone.0093904

**Published:** 2014-04-04

**Authors:** Xiao-Xia Li, Gong-Ping Sun, Jin Meng, Xin Li, Yuan-Xin Tang, Zhen Li, Mo-Fei Wang, Gao-Feng Liang, Xiao-Bo Lu

**Affiliations:** 1 Department of Gastrointestinal Surgery, the Fourth Affiliated Hospital of China Medical University, Shenyang, P.R. China; 2 Department of Toxicology, School of Public Health, China Medical University, Shenyang, P.R. China; National Cancer Center, Japan

## Abstract

**Objective:**

This meta-analysis was performed to evaluate the role of toll-like receptor 4 (TLR-4) in colorectal carcinogenesis.

**Methods:**

The PubMed, CISCOM, CINAHL, Web of Science, Google Scholar, EBSCO, Cochrane Library, and CBM databases were searched from inception through November 1st, 2013 without language restrictions. Odds ratios (ORs) or standardized mean differences (SMD) with their 95% confidence intervals (CI) were calculated.

**Results:**

Fourteen case-control studies met the inclusion criteria for this meta-analysis. A total of 1,209 colorectal cancer (CRC) cases and 1,218 healthy controls were involved in this meta-analysis. Two common polymorphisms (299 A>G and 399 C>T) in the *TLR-4* gene, TLR-4 mRNA and protein expression were assessed. Our meta-analysis results revealed that the *TLR-4* 399 C>T polymorphism might increase the risk of CRC (allele model: OR = 1.77, 95%CI = 1.32∼2.36, *P*<0.001; dominant model: OR = 1.83, 95%CI = 1.32∼2.52, *P*<0.001; respectively). However, we found no correlation between the *TLR-4* 299 A>G polymorphism and CRC risk (all *P*>0.05). A subgroup analysis by ethnicity suggested that *TLR-4* genetic polymorphisms were associated with an increased risk of CRC among Asians (allele model: OR = 1.50, 95%CI = 1.19∼1.88, *P* = 0.001; dominant model: OR = 1.49, 95%CI = 1.16∼1.92, *P* = 0.002; respectively), but not among Caucasians and Africans (all *P*>0.05). Furthermore, our results showed that TLR-4 mRNA and protein levels in CRC patients were higher than those in healthy controls (TLR-4 mRNA: SMD  = 2.51, 95%CI  = 0.98∼4.05, *P* = 0.001; TLR-4 protein: OR  = 4.75, 95%CI  = 1.16∼19.36, *P* = 0.030; respectively).

**Conclusion:**

Our findings provide empirical evidence that TLR-4 may play an important role in colorectal carcinogenesis. Thus, TLR-4 is a promising potential biomarker for the early diagnosis of CRC.

## Introduction

Colorectal cancer (CRC), which includes colon and rectal cancers, is the third most commonly diagnosed cancer in males and the second in females [1], [2]. The symptoms of CRC typically include rectal bleeding and anemia, which are sometimes associated with weight loss and changes in bowel habits [3]. It has been recognized that colorectal cancer is a multifactorial disease caused by complex interactions between environmental and genetic factors [4]. Risk factors for CRC consist of high intakes of fat and alcohol, obesity, smoking and lack of physical exercise [5]. Nowadays, many candidate genes have been identified, such as toll-like receptor 4 (TLR-4), which may be implicated in the genesis of colorectal cancer [6], [7].

TLR-4 belongs to a family of Toll-like receptors (TLRs), which are receptors playing the main role in the recognition of a wide array of pathogens, including viruses, bacteria, protozoa, and fungi [8]. The human *TLR-4* gene is located in chromosomal region 9 (9q32-q33) and consists of four exons and three introns with an overall length of approximately 19 kb [9]. As one of the most actively investigated TLR, TLR-4 has been implicated in signal transduction events induced by the lipopolysaccharide of gram-negative bacteria and its activation in the production of several pro-inflammatory, antiviral and anti-bacterial cytokines [10], [11]. Since TLR-4 is critical in immune and inflammation responses to various bacteria in the intestine [12], single nucleotide polymorphisms (SNPs) in *TLR-4* may decrease the response to bacterial components, impact gut homeostasis, result in the impairment of TLRs activation, and thereby be conducive to the development of several inflammatory diseases including CRC [13], [14]. Furthermore, human CRC cell lines with high rates of microsatellite instability were found to stimulate the activation of TLR-4 through the release of cytokines, and elevate the level of TLR-4 mRNA, thereby inducing the pathogenesis of CRC [15]. Therefore, it was hypothesized that TLR-4 might play important roles in the development and progression of CRC [16]. Additionally, it is also worth noting that numerous studies have been conducted to investigate the potential associations between common polymorphisms in the *TLR-4* gene and CRC risk, especially focusing on two SNPs, (299 A>G, rs4986790 and 399 C>T, rs4986791), which are located in the extracellular domain of TLR-4 [8], [10], [17]. These two important SNPs may alter the amino acid sequence of the TLR-4 protein, disrupting the normal structure of the extracellular domain of the TLR-4 and TLR-4 signaling [14]. The dysregulation of TLR-4 signaling may change the ligand binding and balance between pro- and anti-inflammatory cytokines, creating a pro-inflammatory environment that favors tumor growth, thereby modulating the risk of CRC [10]. However, previous studies have arrived at contradictory results [10], [13], [18]. Consequently, we performed the present meta-analysis to evaluate the exact role of TLR-4 in colorectal carcinogenesis.

## Methods

### Literature search

The PubMed, CISCOM, CINAHL, Web of Science, Google Scholar, EBSCO, Cochrane Library, and CBM databases were searched for relevant articles published before November 1st, 2013 without any language restrictions. The following keywords and MeSH terms were used: [“colorectal cancer” or “CRC” or “colorectal tumor” or “colorectal neoplasm” or “colorectal carcinogenesis” or “colon cancer” or “rectal cancer”] and [“toll-like receptor 4” or “TLR-4” or “toll-4 receptor” or “toll 4 receptor”]. We also performed a manual search to find other potential articles.

### Selection criteria

The included studies had to meet all the following criteria: (1) the study must be clinical cohort or case-control study; (2) the study must relate to the role of TLR-4 in colorectal carcinogenesis; (3) all patients must conform to the diagnostic criteria of CRC; (4) the study must provide sufficient information about *TLR-4* SNP frequencies, mRNA or protein expressions. If the study did not meet the inclusion criteria, it was excluded. When authors published several studies using the same subjects either the most recent or the largest sample size publication was included. The supporting PRISMA checklist is available as supplementary information; see Checklist S1.

### Data extraction

Relevant data were systematically extracted from all included studies by two researchers using a standardized form. The researchers collected the following data: language of publication, publication year, the first author's surname, geographical location, design of study, sample size, the source of the subjects, SNP frequencies, mRNA/protein levels, source of samples, genotyping method, mRNA/protein detection method, etc.

### Quality assessment

Methodological quality was independently assessed by two researchers according to the Newcastle-Ottawa Scale (NOS) criteria [19]. The NOS criteria assigns scores based on three aspects: (1) subject selection: 0∼4; (2) comparability of subject: 0∼2; (3) clinical outcome: 0∼3. Total NOS scores range from 0 to 9 with a score ≥7 indicating high quality. The supporting NOS score criterion is available in Supplement S1.

### Statistical analysis

The STATA version 12.0 (Stata Corp, College Station, TX, USA) software was used for this meta-analysis. We calculated crude odds ratio (OR) with their 95% confidence interval (95%CI) to evaluate the specified relationships. The *Z* test was used to estimate the statistical significance of pooled statistics. The Cochran's *Q*-statistic and *I^2^* test were used to evaluate potential heterogeneity between studies [20]. If *Q*-test showed a *P*<0.05 or *I^2^* test exhibited >50%, indicating significant heterogeneity, the random-effect model was conducted; otherwise the fixed-effects model was used. We also performed subgroup and meta-regression analyses to investigate potential sources of heterogeneity. In order to evaluate the influence of single studies on overall estimates, a sensitivity analysis was performed. We also conducted Begger's funnel plots and Egger's linear regression test to investigate publication bias [21].

## Results

### Characteristics of included studies

**Figure 1:**
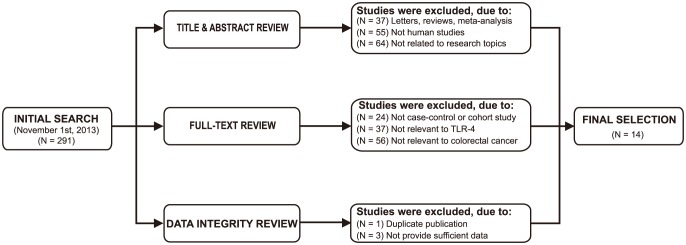
Flow chart of literature search and study selection. Fourteen case-control studies were included in this meta-analysis.

**Figure 2:**
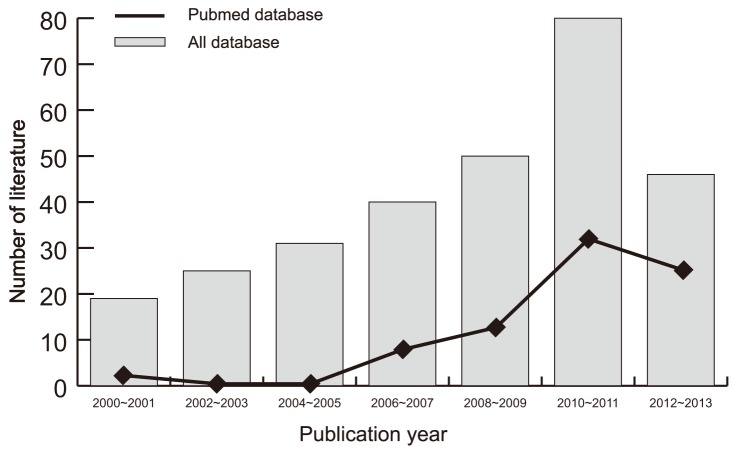
Distribution of the number of topic-related literatures in electronic databases over the last decade.

Initially, the searched keywords identified 291 articles. We reviewed the titles and abstracts of all articles and excluded 156 articles; full texts and data integrity were then reviewed and a further 121 articles were excluded. Finally, 14 case-control studies were included in this meta-analysis [10], [13], [14], [18], [22]–[31]. Publication years of the eligible studies range from 2006 to 2013. Figure 1 shows the selection process of eligible articles. The distribution of the number of topic-related literature in electronic databases over the last decade is shown in Figure 2. A total of 2,427 subjects were involved in this meta-analysis, including 1,209 CRC patients and 1,218 healthy controls. Eleven studies were performed in Asian populations, two studies in Caucasian populations and only one study in an African population. The NOS scores of all included studies were ≥5 (moderate-high quality). We summarized the study characteristics and methodological quality in Table 1.

**Table 1:** Main characteristics and methodological quality of all eligible studies.

First author [Ref]	Year	Country	Ethnicity	Sample size		Gender (male/female)		Age (years)		Marker	Detection method	NOS score
				Case	Control	Case	Control	Case	Control			
Pimentel-Nunes P [13]	2013	Portugal	Caucasian	193	278	123/70	176/102	62.0±7.0	56.0±4.0	299 A>G	PCR-RFLP	7
Omrane I [10]	2013	Tunisia	African	100	140	53/47	45/95	58.2±14.4	56.2±15.7	299 A>G	Direct sequencing	8
										399 C>T	Direct sequencing	8
Dai Q [21]	2012	China	Asian	268	268	-	-	-	-	299 A>G	PCR-RFLP	6
Yang GG [22]	2011	China	Asian	102	87	64/38	52/35	52	50	299 A>G	Direct sequencing	6
										399 C>T	Direct sequencing	6
Boraska Jelavic T [17]	2006	Croatia	Caucasian	89	88	61/28	61/67	61.5±9.5	61.5±9.5	299 A>G	PCR-RFLP	6
										399 C>T	PCR-RFLP	6
Davoodi H [14]	2011	Malaysia	Asian	60	50	30/30	25/25	30∼50	30∼50	299 A>G	PCR-RFLP	6
										399 C>T	PCR-RFLP	6
Nihon-Yanagi Y [28]	2012	Japan	Asian	50	50	22/28		68 (52∼90)		mRNA	RT-PCR	7
Yu YH [30]	2011	China	Asian	62	25	-		-		mRNA	RT-PCR	6
Huang HY [26]	2010	China	Asian	63	63	35/28		-		mRNA	RT-PCR	7
Jin HM [27]	2009	China	Asian	24	24	11/13		67 (35∼86)		mRNA	RT-PCR	8
Tian F [29]	2008	China	Asian	30	30	18/12		64 (51∼82)		mRNA	RT-PCR	8
Hu KF [25]	2013	China	Asian	40	20	27/13		32∼80		Protein	SP	8
Cheng ZL [23]	2012	China	Asian	58	25	38/20		22∼83		Protein	SP	8
Guo YW [24]	2007	China	Asian	70	70	42/28		33∼71		Protein	Flow cytometry	8

Legend: PCR-RFLP - polymerase chain reaction-restriction fragment length polymorphism; NOS - the Newcastle-Ottawa Scale.

### TLR-4 genetic polymorphisms with CRC risk

**Figure 3:**
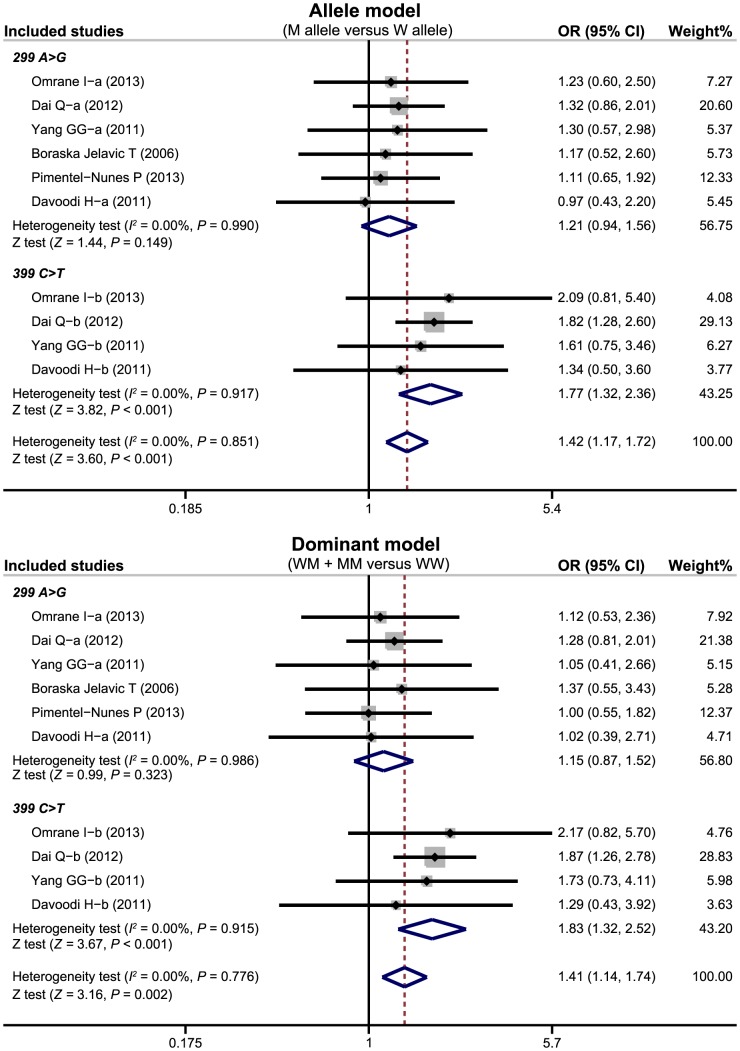
Forest plots for the relationships of *TLR-4* genetic polymorphisms with the risk of colorectal cancer under the allele and dominant models. (a) *TLR-4* 299 A>G; (b) *TLR-4* 399 C>T.

There were six studies focused on the correlation of *TLR-4* genetic polymorphisms with susceptibility to CRC. Our meta-analysis findings on the relationships between *TLR-4* genetic polymorphisms and the risk of CRC are shown in Table 2. The random effects model was conducted due to the existence of significant heterogeneity between studies. Two common polymorphisms in the *TLR-4* gene (299 A>G, rs4986790; 399 C>T, rs4986791) were assessed. Our meta-analysis results revealed that the *TLR-4* 399 C>T polymorphism might increase the risk of CRC (allele model: OR  = 1.77, 95%CI: 1.32∼2.36, *P*<0.001; dominant model: OR  = 1.83, 95%CI: 1.32∼2.52, *P*<0.001; respectively) (Figure 3). Nevertheless, there was no evidence to support any association between the *TLR-4* 299 A>G polymorphism and CRC risk (all *P*>0.05).

**Table 2:** Meta-analysis of the associations between *TLR-4* genetic polymorphisms and colorectal cancer risk.

	M allele vs. W allele			WM + MM vs. WW			MM vs. WW + WM			MM vs. WW			MM vs. WM		
	(Allele model)			(Dominant model)			(Recessive model)			(Homozygous model)			(Heterozygous model)		
	OR	95%CI	*P*	OR	95%CI	*P*	OR	95%CI	*P*	OR	95%CI	*P*	OR	95%CI	*P*
Overall	1.42	1.17∼1.72	<0.001	1.41	1.14∼1.74	0.002	1.85	1.00∼3.44	0.051	1.93	1.04∼3.59	0.038	1.61	0.82∼3.13	0.163
*SNP type*															
299 A>G	1.21	0.94∼1.56	0.149	1.15	0.87∼1.52	0.323	1.65	0.77∼3.54	0.198	1.67	0.78∼3.59	0.187	1.61	0.70∼3.69	0.257
399 C>T	1.77	1.32∼2.36	<0.001	1.83	1.32∼2.52	<0.001	2.32	0.80∼6.72	0.120	2.54	0.88∼7.38	0.086	1.60	0.52∼4.93	0.417
*Ethnicity*															
Africa	1.49	0.84∼2.62	0.173	1.44	0.77∼2.72	0.256	3.63	0.17∼76.40	0.407	3.63	0.17∼76.57	0.407	3.65	0.16∼82.33	0.416
Asians	1.50	1.19∼1.88	0.001	1.49	1.19∼1.92	0.002	2.01	0.96∼4.21	0.065	2.11	1.01∼4.44	0.048	1.68	0.76∼3.72	0.202
Caucasians	1.13	0.72∼1.78	0.539	1.10	0.66∼1.81	0.719	1.33	0.37∼4.82	0.664	1.36	0.40∼4.61	0.625	1.14	0.17∼7.46	0.895
*Genotyping method*															
Non-PCR-RFLP	1.37	0.88∼2.12	0.166	1.26	0.78∼2.04	0.351	2.15	0.60∼7.69	0.238	2.21	0.62∼7.90	0.223	1.85	0.46∼7.38	0.382
PCR-RFLP	1.44	1.16∼1.78	0.001	1.44	1.14∼1.82	0.002	1.77	0.87∼3.60	0.115	1.85	0.91∼3.76	0.090	1.54	0.72∼3.30	0.267
*Sample size*															
Large (n > 200)	1.48	1.19∼1.86	0.001	1.43	1.10∼1.87	0.008	3.02	1.24∼7.38	0.015	3.17	1.30∼7.76	0.011	2.60	1.03∼6.54	0.043
Small (n ≤ 200)	1.26	0.87∼1.83	0.217	1.29	0.84∼1.97	0.246	1.18	0.50∼2.78	0.709	1.21	0.51∼2.87	0.660	0.95	0.36∼2.50	0.916

Legend: W - wild allele; M - mutant allele; WW - wild homozygote; WM - heterozygote; MM - mutant homozygote; OR - odds ratio; 95%CI - 95% confidence interval; SNP - single nucleotide polymorphism; PCR-RFLP - polymerase chain reaction-restriction fragment length polymorphism.

**Figure 4:**
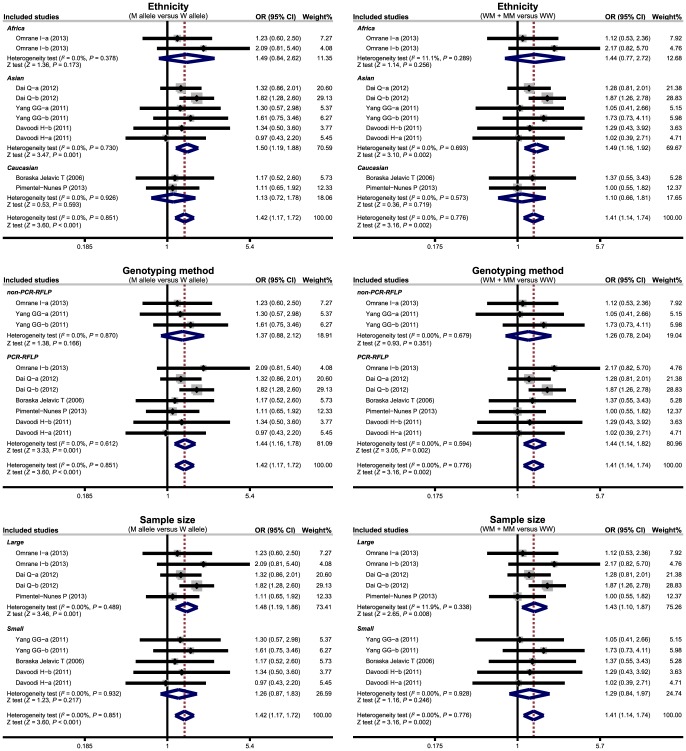
Subgroup analyses by ethnicity and genotyping method of the relationships of *TLR-4* genetic polymorphisms with the risk of colorectal cancer under the allele and dominant models. (a) *TLR-4* 299 A>G; (b) *TLR-4* 399 C>T.

Subgroup analyses were conducted based on ethnicity, genotyping method and sample size to investigate potential sources of heterogeneity. Our findings suggested that genetic polymorphisms in *TLR-4* genes were associated with an increased risk of CRC among Asian populations (allele model: OR  = 1.50, 95%CI  = 1.19∼1.88, *P* = 0.001; dominant model: OR  = 1.49, 95%CI  = 1.16∼1.92, *P* = 0.002; respectively), but not among Caucasian or African populations (all *P*>0.05) (Figure 4). We also found that *TLR-4* genetic polymorphisms were closely linked to CRC risk in the PCR-RFLP subgroup (allele model: OR  = 1.44, 95%CI  = 1.16∼1.78, *P* = 0.001; dominant model: OR  = 1.44, 95%CI  = 1.14∼1.82, *P* = 0.002; respectively) and the large sample-size subgroup (allele model: OR  = 1.48, 95%CI  = 1.19∼1.86, *P* = 0.001; dominant model: OR  = 1.43, 95%CI  = 1.10∼1.87, *P* = 0.008; respectively), but no similar correlations were observed in non-PCR-RFLP or small sample-size subgroups (all *P*>0.05). Our meta-regression analyses suggested that ethnicity was a major source of heterogeneity (as shown in Table 3).

**Table 3:** Univariate and multivariate meta-regression analyses of potential source of heterogeneity.

Heterogeneity factors	Coefficient	SE	*Z*	*P*	95%CI	
					LL	UL
*Publication year*						
Univariate	0.026	0.063	0.42	0.678	−0.097	0.149
Multivariate	0.040	0.097	−0.42	0.678	−0.230	0.150
*SNP type*						
Univariate	0.381	0.197	1.93	0.053	−0.006	0.781
Multivariate	0.376	0.208	1.81	0.071	−0.032	0.783
*Ethnicity*						
Univariate	−0.067	0.143	−0.47	0.638	−0.347	0.213
Multivariate	0.031	0.163	−0.19	0.851	−0.350	0.289
*Genotyping method*						
Univariate	−0.050	0.250	−0.20	0.840	−0.540	0.439
Multivariate	0.137	0.323	0.42	0.672	−0.496	0.770
*Sample size*						
Univariate	−0.161	0.221	−0.73	0.467	−0.595	0.273
Multivariate	−0.284	0.382	−0.74	0.457	−1.032	0.467

Legend: SE - standard error; 95%CI - 95% confidence interval; UL - upper limit; LL - lower limit; SNP - single nucleotide polymorphism.

### Expression of TLR-4 mRNA and protein in colorectal carcinogenesis

**Figure 5:**
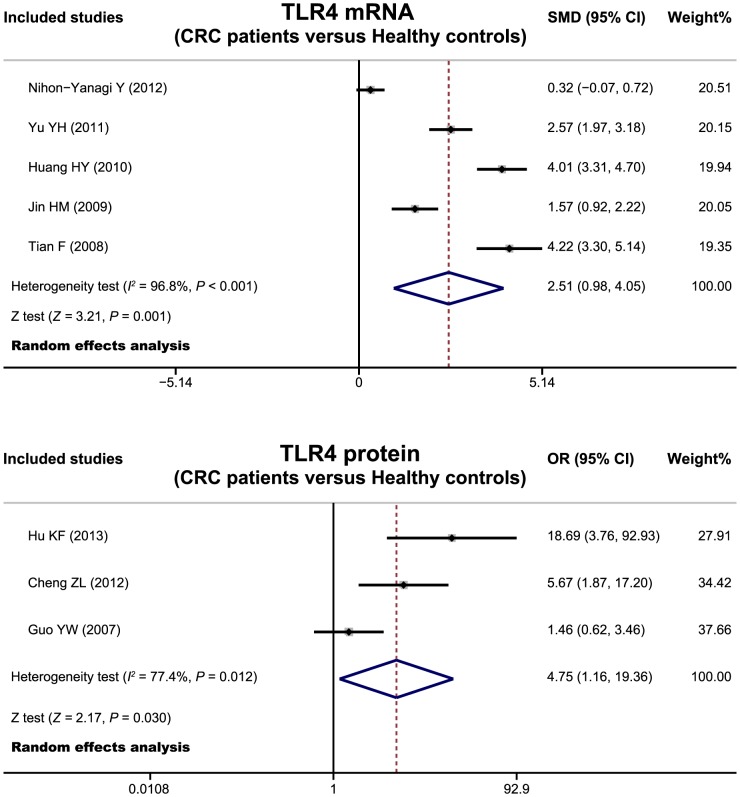
Forest plots for the relationships of TLR-4 mRNA and protein expression with colorectal carcinogenesis.

Five studies evaluated the potential role of TLR-4 mRNA expression in colorectal carcinogenesis. The pooled results of these studies indicated that CRC patients had a higher levels of TLR-4 mRNA than those of healthy controls (SMD  = 2.51, 95%CI  = 0.98∼4.05, *P* = 0.001) (Figure 5). In addition, three studies reported correlations between TLR-4 protein expression and colorectal carcinogenesis. Meta-analysis results also showed a significant difference in TLR-4 protein levels between CRC patients and healthy controls (OR  = 4.75, 95%CI  = 1.16∼19.36, *P* = 0.030).

### Sensitivity analysis and publication bias evaluation

**Figure 6:**
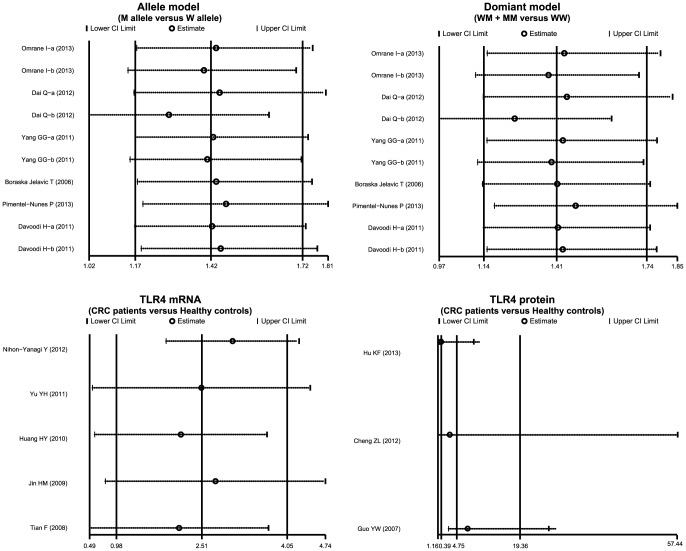
Sensitivity analysis of the summary odds ratio coefficients on the relationships of *TLR-4* genetic polymorphisms, TLR-4 mRNA and protein expression with colorectal carcinogenesis. (a) *TLR-4* 299 A>G; (b) *TLR-4* 399 C>T.

**Figure 7:**
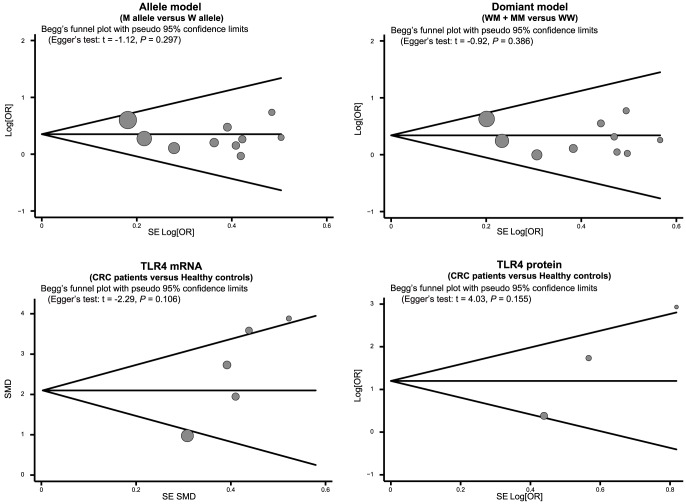
Begger's funnel plot of publication biases on the relationships of *TLR-4* genetic polymorphisms, TLR-4 mRNA and protein expression with colorectal carcinogenesis.

The results of a sensitivity analysis suggested that no single study significantly influenced overall pooled estimates (Figure 6). We also found no evidence of obvious asymmetry in Begger's funnel plots (Figure 7). Egger's test also did not display strong statistical evidence of publication bias (allele mode: *t* = −1.12, *P* = 0.297; dominant model: *t* = −0.92, *P* = 0.386; TLR-4 mRNA: *t* = −2.29, *P* = 0.106; TLR-4 protein: *t* = 4.03, *P* = 0.155; respectively).

## Discussion

TLR-4, a member of the IL-1R (IL-1 receptor) superfamily, shares the cytoplasmic region known as the TIR (Toll/IL-1R) domain [32]. TLRs have been suggested to play a key role in the recognition of pathogens, including protozoa, viruses, bacteria, and fungi, and thus are involved in innate and adaptive immune response processes [33], [34]. In general, TLR-4 is highly expressed in monocytes, macrophages, lymphocytes, and dendritic cells, and can also be observed in many types of epithelial and endothelial cells [35], [36]. It is widely accepted that epithelial cells of the intestine are responsible for regulating the immune response to bacterial antigen, while the sustained activation of the TLR-4 signaling pathway accompanied with an elevated levels of TLR-4 mRNA may stimulate the expression of TLR-4 and in turn act as a critical mechanism for the acquisition of malignant phenotypes by epithelial cancer cells [37]. Consequently, genetic polymorphisms in *TLR-4* may promote the synthesis of proinflammatory cytokines, which are causative factors in the pathogenesis of CRC 38–40. Furthermore, the expression of TLR-4 mRNA and protein has also been postulated to be strongly associated with colorectal carcinogenesis [41].

In order to evaluate the exact role of TLR-4 in colorectal carcinogenesis, we performed a meta-analysis of 14 case-control studies with a total of 1,209 CRC patients and 1,218 healthy controls. Our meta-analysis results indicated that the *TLR-4* 399 C>T polymorphism was significantly associated with the risk of CRC, which suggests that this polymorphism may be involved in the development of CRC. However, we found no evidence of any associations between the *TLR-4* 299 A>G polymorphism and CRC risk, revealing that this polymorphism may not be an important determinant of colorectal carcinogenesis. Although the exact function of *TLR-4* genetic polymorphisms in colorectal carcinogenesis is still not well understood, on possible explanation is that mutations in the *TLR-4* gene could decrease cyclooxygenase-2 (COX-2) expression, which is responsible for increasing commensal bacteria, thereby causing inflammation and leading to CRC [42]. In addition, it is well established that SNPs may contribute to amino acid substitutions altering protein function and may also change transcription factor binding motifs, promoting an alternative translation initiation codon, and thereby resulting in a wild-type transcript down-regulation in the promoter region [43], [44]. In fact, 1196C/T and 896A/G are the most studied genetic variations of *TLR-4.* The nonsynonymous polymorphisms *TLR-4* 1196C/T (major allele C, minor allele T, rs4986791) and *TLR-4* 896A/G (major allele A, minor allele G, rs4986790), which are located in the fourth exon, may affect the extracellular domain of TLR4 and lead to the transitions of cytosine-thymine (C-T) and adenine-guanine (A–G), respectively [8]. In turn, such progressions may cause substitutions in amino acids: isoleucine instead of threonine at 399 position (Thr399Ile) and glycine instead of aspartic acid at 299 position (Asp299Gly) [45]. In this regard, *TLR-4* SNPs of the transmembrane domain may lead to defects in intracellular receptor transfer that prevent the receptor to be converted to the cell surface where it works, and thereby may correlate with disorders in the immune system related to cancer of the intestinal mucosa [41]. A subgroup analysis indicates that *TLR-4* genetic polymorphisms were associated with an increased risk of CRC among Asians, but not among Caucasians or Africans, which suggests that ethnicity might have been a source of heterogeneity. While the molecular basis of ethnic differences in susceptibility to CRC is currently not fully understood, possible sources of such differences could be natural selection and random genetic drift. A meta-regression analysis also confirms that ethnicity might have been a major source of heterogeneity. In addition, we found that TLR-4 mRNA and protein levels in CRC patients were higher than those in healthy controls, revealing that TLR-4 mRNA and protein may be implicated in the development and progression of CRC. These results may be explained by the fact that TLR-4 is a crucial regulator that helps maintain the balance between commensal bacteria in the gut and the mucosal immune system, while the abnormal expression of TLR-4 leads to the breakdown of homeostasis, which may be a key feature in the pathogenesis of CRC [41]. Furthermore, TLR-4 activation may promote the development of CRC by including enhanced COX-2 expression and increased EGFR signaling [46]. All in all, our findings were consistent with previous studies that TLR-4 may play an important role in colorectal carcinogenesis and may be a potential biomarker for the early diagnosis of CRC.

As the first meta-analysis focusing on the role of TLR-4 in colorectal carcinogenesis, our study has some limitations. First, our results lacked sufficient statistical power to assess the exact roles of *TLR-4* SNPs, mRNA and protein expression in colorectal carcinogenesis due to relatively small sample sizes. Second, meta-analysis is a retrospective study that may lead to subject selection bias, which thus may have affected the reliability of our results. Third, our meta-analysis failed to obtain original data from included studies, which may have limited a further evaluation of the potential role of TLR-4 in the development and progression of CRC. A fourth limitation of this article is the fact that the significant *P*-values reported were driven by one single study with a small sample size, which may seriously affect the accuracy and comprehensiveness of this result. In addition, the inclusion criteria of cases and controls were not well defined in all included studies, which might also influence our results. Most importantly, this study was based on a small number of studies with very small sample sizes, especially for epidemiological research, which constrains the general applicability of our findings.

In conclusion, our findings provide empirical evidence that TLR-4 may play an important role in colorectal carcinogenesis. Thus, TLR-4 is a promising potential biomarker for the early diagnosis of CRC. However, due to the limitations mentioned above, more research studies with larger sample size are needed to achieve a more precise statistical analysis.

## Supporting Information

Checklist S1 **PRISMA Checklist.**
(DOC)

Supplement S1 **The Newcastle-Ottawa Scale for assessing methodological quality.**
(DOC)

Flow Diagram S1 **Flow chart of literature search and study selection.** Fourteen case-control studies were included in this meta-analysis.
(DOC)
