# Supplementary material for: Role of Toll-Like Receptor 4 in Colorectal Carcinogenesis: A Meta-Analysis
Source: PLoS One. 2014 Apr 4;9(4):e93904. doi: 10.1371/journal.pone.0093904 (PMC3976338; doi:10.1371/journal.pone.0093904)
Supplement: Flow Diagram S1 — Flow chart of literature search and study selection. Fourteen case-control studies were included in this meta-analysis. (DOC) [file pone.0093904.s003.doc]

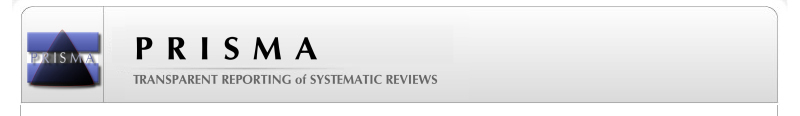
**PRISMA 2009 Flow Diagram**

**Screening**

**Included**

**Eligibility**

**Identification**

Records identified through database searching
(n = 291)

Additional records identified through other sources
(n = 0)

Records after duplicates removed
(n = 290)

Records screened
(n = 290)

Records excluded
(n = 156)

Full-text articles assessed for eligibility
(n = 134)

Full-text articles excluded, with reasons
(n = 117)

Studies included in qualitative synthesis
(n = 17)

Studies included in quantitative synthesis (meta-analysis)
(n = 14)
